# First evidence of transovarial transmission of Kyasanur Forest disease virus in *Haemaphysalis* and *Rhipicephalus* ticks in the wild

**DOI:** 10.1186/s13071-024-06643-5

**Published:** 2025-01-17

**Authors:** Sarah J. Burthe, Bhimanagoud Kumbar, Stefanie M. Schäfer, Bethan V. Purse, Abi T. Vanak, Natrajan Balakrishnan, Richard Hassall, Subhash L. Hoti, Darshan Narayanaswamy, Santoshkumar Potadar, Mujeeb Rahman, Mohammed Mudassar Chanda

**Affiliations:** 1 UK Centre for Ecology & Hydrology, Penicuik, EH26 0QB UK; 2 ICAR-National Institute of Veterinary Epidemiology and Disease Informatics, Bengaluru, India; 3 UK Centre for Ecology & Hydrology, Wallingford, OX10 8BB UK; 4 Ashoka Trust for Research in Ecology and the Environment, Bengaluru, India; 5 ICMR-Vector Control Research Centre, Puducherry, India; 6 Virus Diagnostic Laboratory, Department of Health and Family Welfare Services, Government of Karnataka, Shimoga, India; 7 ICMR-National Institute of Traditional Medicine, Belgaum, India

**Keywords:** Monkey Fever, Zoonotic diseases, Tick-borne pathogen, Emerging infection

## Abstract

**Background:**

Kyasanur forest disease virus (KFDV) is a tick-borne flavivirus causing debilitating and potentially fatal disease in people in the Western Ghats region of India. The transmission cycle is complex, involving multiple vector and host species, but there are significant gaps in ecological knowledge. Empirical data on pathogen-vector-host interactions and incrimination have not been updated since the last century, despite significant local changes in land use and the expansion of KFD to new areas. Mathematical models predict that transovarial transmission, whereby adult female ticks pass KFDV infections to their offspring, plays an important role in the persistence of KFD, but this has not been shown in the wild. Here we set out to establish whether transovarial transmission of KFDV was occurring under natural field conditions by assessing whether host-seeking larvae were positive for KFDV.

**Methods:**

Ticks were sampled by dragging and flagging across a broad range of habitats within the agro-forest matrix at 49 sites in two districts: Shivamogga, Karnataka and Wayanad, Kerala (September 2018-March 2019), and larvae were tested for KFDV by PCR.

**Results:**

In total, larval ticks from 7 of the 49 sites sampled tested positive for KFDV, indicating that transovarial transmission is occurring. Of the 13 KFDV-positive larval samples, 3 came from around houses and gardens, 5 from crops (3 from harvested rice paddy and 2 from areca plantation), 1 from teak plantation and 4 (2 from 1 transect) from forests. Five different tick species were found to have KFDV-positive larvae: *Haemaphysalis spinigera, H. bispinosa, Rhipicephalus annulatus, R. microplus* and an unidentifiable species of *Haemaphysalis* (no close match in GenBank).

**Conclusions:**

Our empirical confirmation of transovarial transmission has important implications for understanding and predicting KFD dynamics, suggesting that ticks may act as a reservoir for KFDV. Moreover, small mammals and cattle may play crucial roles in transmission if small mammals are the main hosts for larvae infected via transovarial transmission, and cattle support large numbers of infected female adult ticks. This first report of transovarial transmission of KFDV, and within a hitherto undescribed range of vectors and habitats, will help disease managers improve KFD surveillance and mitigation strategies, ultimately leading to communities becoming more resilient to the risk of this tick-transmitted disease.

**Graphical Abstract:**

**Figure:**
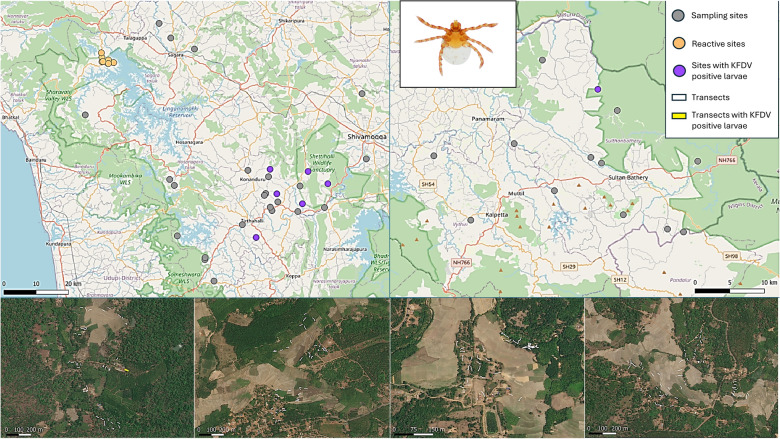

**Supplementary Information:**

The online version contains supplementary material available at 10.1186/s13071-024-06643-5.

## Background

Tick-borne pathogens have significant impacts on livestock and human health and welfare worldwide. Throughout North America, Europe and Asia, pathogens transmitted by ticks are the most common vector-borne diseases in humans [1]. Moreover, in some regions, the emergence, spatial distribution and prevalence of tick-borne diseases are increasing under climate and land-use change [1–3]. The design and implementation of effective control against tick-borne diseases are often limited by a poor ecological evidence base, notably by a lack of knowledge about the vectors, host species and processes that underpin transmission and lead to spillover into human populations [4, 5]. This is particularly so for tick-borne infections affecting poor and marginalised populations in low resource settings, where such diseases receive less attention and funding for research and mitigation [6, 7]. Elucidating the transmission cycle of tick-borne-diseases is highly challenging because it often involves a complex range of vector and vertebrate hosts and potentially three different mechanisms by which tick vectors acquire the pathogens [8]. Systemic transmission of pathogens occurs when susceptible ticks feed on a host with a systemic infection. Non-systemic or co-feeding transmission occurs when a susceptible tick acquires a pathogen by feeding in close proximity to an infected tick, without systemic infection occurring in the host [9]. Finally, transovarial transmission occurs when the pathogen is transmitted from the adult female tick to her offspring. Identifying which modes of transmission are operating within a pathogen-vector system is vital for understanding pathogen-vector-host dynamics and identifying the seasonal and spatial zoonotic hazards acting as potential sources of harm to humans within a landscape [8, 10].

Kyasanur forest disease virus (KFDV) is a tick-borne flavivirus causing debilitating and potentially fatal haemorrhagic disease in people in the Western Ghats region of southwest India, with approximately 500 cases per annum and up to 10% mortality in unvaccinated people [11]. Historically, since Kyasanur forest disease (KFD) was first described in 1957, human cases were restricted to a small number of districts in Karnataka state [11, 12]. Since 2005, human cases of KFD have increased, with a recent dramatic spread to neighbouring states of Goa, Tamil Nadu, Maharashtra and Kerala [13–15]. The disease primarily affects low-income rural forest communities, such as small-holder farmers, plantation and forestry workers, and tribal groups reliant on harvesting of non-timber forest products [16–19]. KFD has been highlighted as a major health issue in the region, with 69% of respondents to household surveys (smallholder farmers and tribal groups; *n* = 227) reporting that they felt vulnerable to the impact of KFD on their livelihoods [20]. As well as infecting humans, KFDV has a broad vector and host range. The transmission cycle, which was largely ascertained from empirical data collected in the 1960s and 1970s, is complex, involving multiple vector and host species. India has a high diversity of Ixodid tick species. The *Haemaphysalis* genus is particularly species-rich, comprising 42 different taxa [21], amongst which 9 species have been previously incriminated in KFDV transmission, alongside several other species of the *Ixodes*, *Rhipicephalus* and *Dermacentor* genera [11]. A broad range of vertebrate hosts have been considered to be KFDV reservoirs, including wild rodents and shrews, bats, monkeys and some birds (reviewed in [11]). However, significant gaps in knowledge of the ecology of the KFD system have been identified [5, 22], including a lack of empirical data on the involvement of cattle in maintaining and moving adult tick populations and facilitating co-feeding transmission and the role of small mammals versus primates as reservoir hosts. Spillover of KFDV to humans has been widely linked to deforestation [23–25] and the replacement of moist evergreen forest with rice cultivation and plantations [11, 23], but the mechanisms underpinning this linkage are not known. Empirical data on pathogen-vector-host interactions and incrimination have not been updated since the last century, despite significant degradation and reduction in forest extent [26] and the expansion of KFD infections to new areas [5, 27].

Recent mathematical models exploring the ecological processes influencing the maintenance of KFD predicted that transovarial transmission, whereby adult female ticks pass KFDV infections to their offspring, plays an important role in the persistence of KFD, alongside systemic transmission particularly amongst small mammal and bird hosts [22, 28]. There is also some experimental evidence from laboratory studies that transovarial transmission occurs in *Haemaphysalis spinigera* and *Ixodes petauristae* tick species [29, 30]. However, both sets of experiments involved unnatural conditions, with inoculation of high concentrations of passaged virus in vectors and hosts. Also, in the case of *H. spinigera*, transovarial transmission was only recorded in larval progeny of adult female ticks that were inoculated with KFDV directly into the haemocoele and not in larval progeny of adult female ticks that had acquired the infection by feeding on KFDV-infected chicks as immatures [30]. To the best of our knowledge, transovarial transmission has not been observed in non-laboratory settings with no prior reports of wild-caught questing larvae having been found to be positive for KFDV. Therefore, our present study set out to establish whether transovarial transmission of KFDV was occurring under natural field conditions by assessing whether host-seeking larvae collected across a broad range of habitats in the Western Ghats were positive for KFDV.

## Methods

### Study sites

**Fig. 1:**
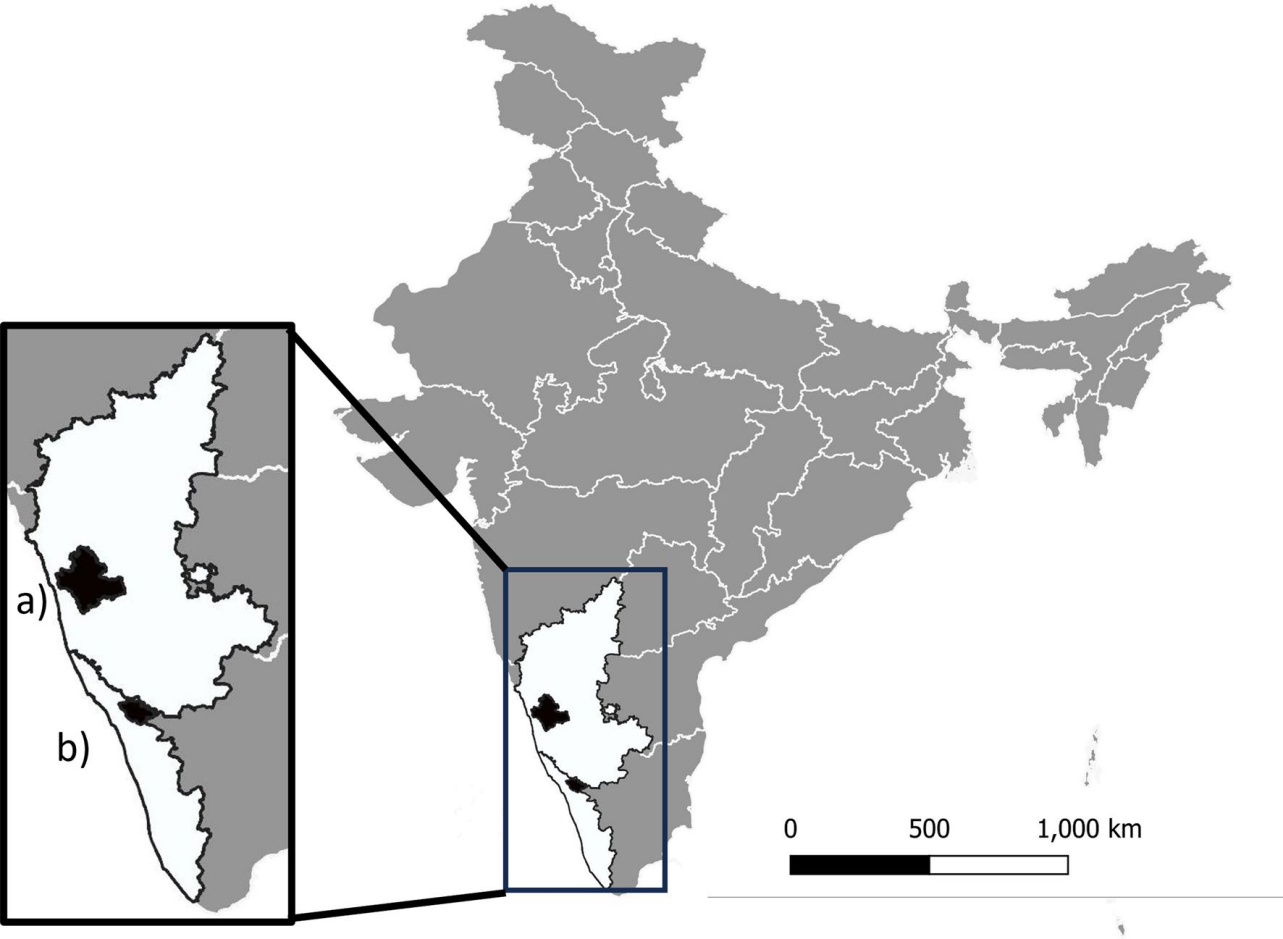
Map of India showing the locations of the two study districts (indicated in black): **a** Shivamogga district in Karnataka state and **b** Wayanad district in Kerala state

Ticks were sampled at 41 village sites across Shivamogga district in Karnataka and Wayanad district in Kerala between September 2018 and April 2019 (Fig. 1). Sampling was undertaken once at each site between September and March to coincide with the main period of human KFD cases and peak timing of tick abundance [17, 31]. Although historically restricted to several districts within Karnataka, since first reported in 1957, human cases have been increasing in numbers and locations within the district [15], whilst cases in Wayanad were first reported in 2014 [27]. Sites were selected according to two criteria: proximity of the village to large natural or semi-natural forest patches (which have been found previously to support a diverse range and high abundance of tick species [21, 31]) and the number of human KFD cases in the 3 years prior to tick sampling (see [19] for details). Large forest patches were those that were at least 0.54 km^2^ in size (at least 6 cells of the land use land cover map), and proximity was calculated as the minimum distance between site coordinates and the nearest large forest patch. Additionally, eight sites in the Shivamogga district were reactively sampled for ticks in sites where human cases were reported during the months of the sampling campaign.

A range of habitats that could potentially support ticks were surveyed at each site: transects were sampled around the houses and gardens, within the dominant agriculture (mainly rice, banana, coffee), within plantation forest (primarily areca nut and teak) and within natural or semi-natural forest. There is currently a lack of empirical evidence on tick distributions across the broad range of habitats, other than forest, within the agro-forest matrix (identified as a research priority in [5]). The results from our stratified ecological surveys across this agro-forest matrix will help address this knowledge gap and will be reported in a subsequent publication.

A detailed land cover map was developed for the study districts and is published in an ISI paper and as a dataset [32]. These data enable estimation of the differential composition of the agro-forest matrix in the two focal districts. In the Shivamogga district, in which 36 of the sites were situated, the land use was made up of 18% evergreen forest, 16% moist evergreen forest, 16% dry deciduous forest, 10% mixed plantation (arecanut, coconut, rubber and eucalyptus plantation), 11% cropland including paddy, 21% fallow land and only 1% built-up areas. In Wayanad district, in which 13 sites were situated, the land use comprised 15% evergreen forest, 15% moist evergreen forest, 7% dry deciduous forest, 46% mixed plantation, 3% teak and 3% tea plantation, 2% cropland including paddy, 3% fallow land and 1% built-up areas.

### Tick larvae collection

Questing ticks were collected using dragging and flagging methods [33], depending on the complexity and structure of the dominant vegetation. Ten 20-m transects were sampled per vegetation type at each site. Dragging was undertaken if the transect comprised vegetation with low height, such as grazed grass, harvested paddy or leaf litter on the forest floor. The drag cloth (1 m × 1 m) was dragged slowly along two 10-m sections of the transect. Flagging was undertaken where ground vegetation had a height of > 20 cm and was structurally complex. Flagging consisted of 10 wide sweeps of the flag (50 cm × 50 cm) across a 5 × 5-m area at the start and end points of the transect. Ticks were removed from the drag cloth after each of the 10-m drags and each of the two sampling sweeps of the flag per transect using fine-tipped forceps. All ticks were placed in 0.5-ml or 1.5-ml tubes prefilled with 200–500 µl of AVL buffer (QIAGEN GmbH) to render the specimen non-infectious for further handling in the laboratory [34]. Ticks were stored in AVL buffer and frozen at − 80 °C until processing. Larvae from each drag or flag were pooled together within one tube. Nymphs and adults were stored individually in separate tubes. Larvae were never stored in the same tubes as nymphs or adults. Due to the time-consuming nature of removing large numbers of larval ticks from the cloths, only the first 25 larvae were removed and stored in tubes. Cloths were then cleaned of ticks by using sticky tape before further drags or flags were undertaken. Here, we just present results for larval ticks. Findings on KFDV infection prevalence and habitat associations of the nymphal and adult tick species sampled will be the subject of subsequent publications.

### Tick identification

To test larval ticks for the presence of KFDV, homogenisation and nucleic acid extraction necessitated the destruction of the tick specimen. Therefore, immediately prior to processing, larval ticks were photographed at 5 × magnification using a stereomicroscope (Zeiss^™^). Photographs were used for identification of larval ticks to genus level using standard morphology-based taxonomic keys [21, 35, 36]. Photographs were also used to ascertain that the questing larvae had not ingested any blood by comparing the body shape and content of the drag- and flag-sampled larvae with those of larvae that had been removed from hosts.

Identification of ticks via morphology can be extremely challenging, particularly for larvae [37, 38], and we therefore used molecular methods to delineate species. We used a molecular taxonomy approach considered to be the best choice for tick species identification [39], based on generating sequence data of the standard DNA barcoding cytochrome oxidase I region (primer details given in Additional file: Table S1) [40].

Processing of ticks took place between 2 and 24 months post-collection. Larval ticks were transferred to a DNase and RNase-free 2-ml tube (Tarsons) and homogenised using a tissue lyser II (QIAGEN GmbH) using 5-mm beads. In cases where more than one larva was present in a sample, larvae were pooled unless initial morphological examination indicated that the sample contained larvae of more than one genus, in which case larvae were handled separately. The homogenised larval samples were then processed for total nucleic acid extraction according to the manufacturer's protocol using a QIAamp Viral RNA Mini Kit (QIAGEN GmbH).

Using the standard primers published by [40], a 710-bp region of the cytochrome oxidase I (COI) gene was amplified by PCR (sees Additional file: Table S1). Three microlitres of DNA template was used in a 50-µl PCR reaction solution that contained 25 µl DreamTaq Green PCR Master Mix (2X) (Thermo Scientific), 10 pmol each of both forward (LC01490) and reverse (HC02198) primers and 20 µl of Nuclease Free water (molecular grade). PCRs were run on a BIO-RAD T100 Thermal Cycler and under the following temperature conditions: an initial denaturation step at 95 °C for 3 min was followed by 35 cycles at 95 °C for 30 s, 50 °C for 30 s, 72 °C for 45 s and a final extension step at 72 °C for 10 min.

Successful amplification of the COI fragment was verified by gel electrophoresis, separating the PCR products on an ethidium bromide-stained 1% agarose gel in 1xTAE buffer and using a GeneRuler 100 bp plus DNA Ladder (Thermo Scientific) as a molecular marker. PCR samples with fragments of the correct size of ca. 710 bp were sent for purification and bidirectional Sanger sequencing to an external company (Eurofins, India).

### Detection of KFDV in tick larvae

Testing of larval samples for KFDV was undertaken by a nested RT-PCR assay targeting the virus’ NS5 gene and broadly following the protocols detailed by [41]. Briefly, cDNA was prepared using the RevertAid RT Reverse Transcriptase kit (Thermo Scientific), according to the manufacturer's protocol. Nested PCR was performed using DreamTaq Green PCR Master Mix (2X) (Thermo Scientific) with 10 pmol of each primer, 3 μl of cDNA and nuclease-free water up to a 25 μl reaction volume.

The first round PCR, using primer set KFDNS5 3S and KFDNS5 3R, was expected to lead to PCR products of 756 bp in length and the second round PCR, using the nested primer set KFDNS5 4S and KFDNS5 4R, to amplicons of 355 bp in size (Additional file: Table S1).

Both the first and second (nested) rounds of PCR amplifications were carried out on a BIO-RAD T100 Thermal cycler and followed the same optimized and modified temperature profile; an initial denaturation step at 95 °C for 5 min was followed by 35 cycles each for 30 s at 95 °C, 30 s at 50 °C, 72 °C for 45 s and a final extension step at 72 °C for 10 min. Then, 2 μl of the first round PCR product was used as template for the second round of this nested PCR. One negative control and one positive control were included with every batch of samples run. The positive control used was extracted KFDV RNA from a known human KFD-positive case obtained from the ICMR-National Institute of Traditional Medicine. Successful amplification of the NS5 fragments was verified by gel electrophoresis (as described above). KFD-positive PCR products were sent for purification and bidirectional Sanger sequencing for further confirmation (Eurofins, India).

### Confirmation of blood meal status

Larval ticks sampled by drag or flag from the environment are in the process of questing, i.e. looking for a suitable host for the first time and should not yet have fed on a host. To confirm that assumption, a blood meal analysis assay, aimed at detecting any evidence of prior ingestion of host blood, was carried out. The total nucleic acid extracted from larval homogenates was subjected to a PCR using a set of vertebrate-universal primers (L14724_hk3 and H15915_hk3, as described in [42, 43]; Additional file: Table S1) that target a section of the mitochondrial cytochrome B gene. Using a 25-µl PCR reaction volume, 3 µl of DNA template was added to 12.5 µl DreamTaq Green Master Mix (Thermo Scientific), 10 pmol of each primer and 8.5 µl of nuclease-free water. PCR was carried out using BIO-RAD T100 Thermal cycler (BIO-RAD). The PCR program consisted of an initial denaturation step at 95 °C for 3 min, followed by 40 cycles at 95 °C for 30 s, 50 °C for 30 s, 72 °C for 1 min and a final extension step at 72 °C for 10 min. One negative control and one positive control containing vertebrate DNA were included in the batch of samples. The amplicons were visualized by gel electrophoresis on 1% agarose gels, as described above.

## Results

**Fig. 2:**
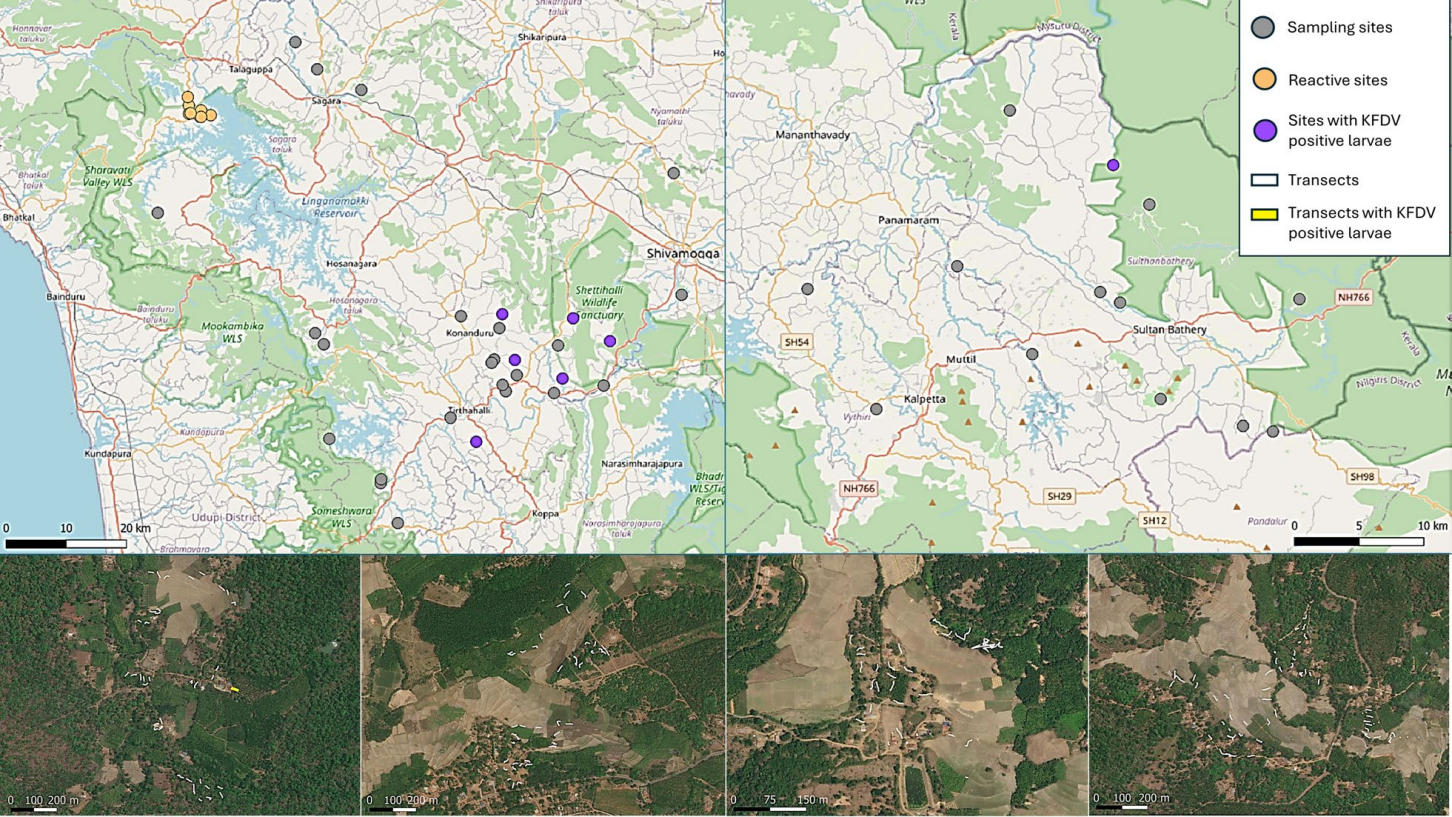
Maps of the study sites across the two districts in the Western Ghats. The upper panel shows locations of sites in Shivamogga district in Karnataka (left side) and Wayanad in Kerala (right side). The four lower panels show four examples of sites indicating tick transects. Basemap data from OpenStreetMap. Satellite basemap data from ESRI World Imagery (sources: Esri, DigitalGlobe, GeoEye, i-cubed, USDA FSA, USGS, AEX, Getmapping, Aerogrid, IGN, IGP, swisstopo and the GIS User Community)

In total, 1495 transects were sampled for ticks across 49 sites, 13 of which were in Kerala and 36 in Karnataka state. Of the 36 sites in Karnataka, 8 were sampled reactively (hereafter reactive sites) in response to reports of current human KFD cases (see Table 1; Fig. 2). On average for the 41 sites systematically sampled, 3.6 (± 1.0 standard deviation, SD) different habitats were sampled for ticks per site with an average of 34.5 (± 9.2 SD) transects per site. Reactive sites were sampled less intensively with an average of 1.6 (± 1.1 SD) habitats sampled per site and an average of 9.9 (± 13.4 SD) transects per site.

**Table 1:** Number of transects sampled for ticks across the 49 sites in Karnataka and Kerala states between September 2018 and March 2019

Habitat type	Total number of transects
Vicinity of houses and gardens	346
Forests (natural/semi-natural dry or moist deciduous or mixed deciduous and evergreen)	270
Rice paddy	304
Open, non-cultivated land (meadows, scrub or fallow land)	113
Areca nut plantation	288
Coffee or tea plantation	57
Teak or silver oak plantation	50
Other plantations (maize, banana, acacia, coconut, eucalyptus, rubber)	67

In total, 2730 larvae were collected. Each transect comprised two potential tick samples (either two 10-m drags per transect or two flagging points per transect), and larval ticks were collected from 771 samples representing 528 transects, with 335 of these samples comprising single larvae and 436 samples containing multiple larvae. We report the number of larvae sampled per transect. In total, 13 larval tick samples collected from 12 transects (2.3% of total transects with larvae) were found to be positive for KFDV.

KFDV-positive larvae were found in 7 of the 41 sites that were systematically sampled for ticks. No positive larvae were found in the eight reactive sites (sites with active cases of human KFD). Five of the KFDV-positive larval ticks were found at a single site (Hallibailu in Shivamogga district) and two from both Demlapura and Tanikal in Shivamogga, with single positive larvae each from a further five sites (Table 2). Of the 343 transects across 13 sites in Wayanad, Kerala, only one transect had a KDFV-positive larva (0.3% of transects with larvae). Six of 35 sites sampled in Shivamogga, Karnataka, had transects with KFDV-positive larval ticks with 10 transects (1 transect had two positive larval pools) positive from a total of 1073 transects (0.9% of transects).

**Table 2:** Species of tick larvae found to be positive for KFDV during sampling of the environment

Species	No. larvae in sample	Site	State	Habitat type
*Haemaphysalis* sp. (no COI barcode)	2	Poothadi	Kerala	Teak plantation
*Haemaphysalis bispinosa*	1	Talale	Karnataka	Rice paddy (harvested)
*Rhipicephalus microplus*	1	Tanikal	Karnataka	Rice paddy (harvested)
*Haemaphysalis spinigera*	1	Tanikal	Karnataka	Arecanut plantation
*Haemaphysalis spinigera*	1	Demlapura	Karnataka	Rice paddy (harvested)
*Haemaphysalis spinigera*	1	Hallibailu	Karnataka	Dry deciduous forest
*Rhipicephalus microplus*	5	Hallibailu	Karnataka	Dry deciduous forest
*Rhipicephalus microplus*	2	Hallibailu	Karnataka	Dry deciduous forest
*Rhipicephalus microplus*	9	Hallibailu	Karnataka	Dry deciduous forest
*Haemaphysalis bispinosa*	6	Hallibailu	Karnataka	Arecanut plantation
*Rhipicephalus annulatus*	1	Sasithota	Karnataka	Around house and gardens
*Rhipicephalus annulatus*	1	Kunaji	Karnataka	Around house and gardens
*Haemaphysalis* (no close match in GenBank)	4	Demlapura	Karnataka	Around house and gardens

The number of larvae in the sample tested column indicates whether a single larva was positive for KFDV or whether the sample comprised a pool of multiple larvae

Of the 13 KFDV-positive larval samples, 3 had been collected from transects in the vicinity of houses and gardens, 5 came from transects in and around crops (3 from harvested rice paddy and 2 from areca nut plantation), 1 was from a forest plantation (teak), and the remaining 4 (2 from one transect) were sampled from dry deciduous semi-natural or natural forests (Table 2; Additional file: Table S2). The 13 KFDV-infected larvae were identified as four different tick species: *Haemaphysalis spinigera, H. bispinosa, Rhipicephalus annulatus* and *R. microplus* (Table 2). However, two larval tick pools could only be identified to genus level, both *Haemaphysalis*. One larval pool produced poor quality sequences, rendering species identification via molecular methods impossible. One larval pool had good quality sequence information but, whilst it seems to fall within the *Haemaphysalis* clade, its COI DNA barcode is < 92% identical to any reliable GenBank or BOLD database submissions. Further work is needed to identify these specimens. Thus, in total we found five species of KFDV-positive questing larvae (see Additional file: Table S3 for details of numbers of negative larvae per species and Fig. S1 for phylogenetic information).

None of the 13 KFDV-positive larval tick samples collected from the dragging and flagging sampling of the environment showed evidence of having had consumed a blood meal. All were negative for vertebrate DNA based on blood meal analysis focusing on the mitochondrial *cytb* gene. Moreover, none showed any morphological evidence of engorgement compared with larval ticks removed from host animals in a partially fed state (Additional file: Fig. S2 and S3).

## Discussion

Here, we show, for the first time to our knowledge, evidence of transovarial transmission of KFDV in *Haemaphysalis* and *Rhipicephalus* ticks in a natural field setting and in multiple sites across two states in the Western Ghats region of India, an area where KFD has substantial impacts on human health. Detection of transovarial transmission provides important support to the theoretical findings from modelling studies that transovarial transmission is necessary, along with systemic transmission, for the persistence of KFD in this region [22, 28]. Empirical confirmation of transovarial transmission has important implications for understanding and predicting KFD dynamics, suggesting that ticks may act as a reservoir for KFDV. Moreover, small mammals and cattle may play a crucial role in transmission dynamics if small mammals are the main hosts infected by larvae infected via transovarial transmission and if cattle support large numbers of infected female adult ticks.

KFDV infections were found in questing larval ticks from two genera, representing at least five different species (one larva could only be identified to genus level and one is an unknown *Haemaphysalis* species): *Haemaphysalis spinigera, H. bispinosa, R. annulatus* and *R. microplus*. Of these species, *H. spinigera* and *H. bispinosa* have previously been incriminated as being vectors for KFDV [44]. Although ticks from the genus *Rhipicephalus* have also been incriminated as being vectors for KFDV, previous studies have identified *R. haemaphysaloides* as a KFDV vector [45] and not, to the best of our knowledge, *R. annulatus* and *R. microplus.* Both *Haemaphysalis* and *Rhipicephalus* ticks have been found previously on a wide range of hosts, including small mammals [46] and livestock [47]. *Haemaphysalis bispinosa* and *R. annulatus*, common tick species on livestock in this area [47, 48], appear capable of transovarial transmission of pathogens. Eggs and unfed larvae reared from adult female ticks of these two species removed from livestock tested positive for bacterial pathogens and parasites *Anaplasma* spp., *Rickettsia* spp. and *Babesia* spp. [48].

Although our study confirmed that transovarial transmission was occurring under field conditions, further work is needed to estimate KDFV prevalence rates and hence the infection risk to animals and to humans from infected tick larvae. Our study was only able to collect a proportion of larvae from drag or flag cloths, and larvae were sometimes pooled when testing for KFDV. Pathogen prevalence in unfed larvae have been estimated successfully in studies of other tick-borne pathogens by assessing the proportion of positive larvae within a nest. For example, larval pathogen prevalence ranged between 2.5% and 97.5% for *Rickettsia* spp., between 2.5% and 82.5% for *Borrelia* spp. and 2.5% and 8.3% for *Anaplasma* spp. in larval nests of *Ixodes* spp. of ticks sampled from dragging and flagging in Germany [49].

By combining laboratory-estimated rates of efficiency of KFDV transmission from adult ticks to eggs (range 0.01–0.1 [29, 30, 45]) and the low expected survival rates from egg to larval ticks (range 0.05–0.25 [28]), we can estimate that for every 1090 eggs laid (average adult fecundity) by adult female ticks, at best, 109 would be infected transovarially, and only 27 (2.5%) of these would survive to become infected larvae. We identified low numbers of KFDV-positive larvae in the environment, suggesting even lower rates of transovarial transmission than expected from the parameters above, at least in the year of our study. We detected KFDV-infected larvae in 7 of the 41 villages systematically sampled and in 0.3% of transects in Wayanad and 0.9% of transects in Shivamogga. Such rates seem low, given that modelling predicts that transovarial transmission and systemic transmission are predicted to contribute approximately equally to maintaining KFD infection in areas where KFDV is present and that predicted R0 has a high sensitivity to the rate of transovarial transmission [22]. It is possible that our KFDV assays are failing to detect positive larvae, given their small size and the potentially small amount of viral RNA present, potentially leading to underestimates of the levels of transovarial transmission. However, KFDV detection methods were optimised in our study from the original methods published in [41]. Previous studies have failed to find KFDV-positive questing larvae despite extensive surveying for ticks, such as [31, 47], and further investigation into the sensitivity of detection methods would be helpful. Despite the wide range of habitats and sites sampled, our study was also limited to sampling ticks within a single season in 1 year. Given that there is clear temporal variability in KFD risk, with human and primate cases of KFD varying significantly between years [50, 51], further work is urgently needed to understand whether transovarial transmission rates might vary between tick species, habitats and years, and why, and the relative importance of transovarial transmission versus systemic transmission under these different conditions.

Our findings have important implications for KFD surveillance and management. First, current public health guidance [52] suggests that infected nymphs are the tick life stage that poses by far the greatest risk for humans. However, if transovarial transmission is occurring, then infected host-seeking larvae may also present an exposure risk to people. Similarly, current management recommendations and surveillance strategies assume that humans are only at risk of exposure to infected ticks in forest habitats, yet we could demonstrate clearly that infected larvae are present across multiple habitat types, even around houses and gardens. In areas with KFD cases, educating people about the risks posed by tick bites across a range of habitats is needed. Multi-lingual, educational material, tailored for local communities, can be found on the IndiaZooRisk and MonkeyFeverRisk project websites (https://monkeyfeverrisk.ceh.ac.uk/ and https://indiazoorisk.ceh.ac.uk/). We also recommend that tick surveillance methods should be more standardised, rigorous and stratified by habitat (extending beyond forests) to build knowledge of tick vector habitat associations and to better assess variation in rates of transovarial transmission in space and time (using better optimised KFDV detection methods). Identification of hotspots of infected unfed larvae would be useful for identifying areas at immediate risk of human exposure to infected larvae and at risk of infection from infected nymphs in the following season. However, given the low numbers of infected host-seeking larvae detected, it is also recommended that surveillance be undertaken to look at infection rates in adult female ticks removed from livestock in areas where humans are at risk of KFD. Cattle are known to support high numbers of ticks, including adult ticks, and may play an important role in amplifying tick populations and dispersing them across habitats. Modelling work has shown that the spatial risk of human KFD cases is associated with the density of cattle in areas long affected by KFD in Shivamogga district [23] and that cattle density is important for the maintenance of KFD [22]. Surveillance of adult female ticks would facilitate estimation of risk through transovarial transmission in subsequent seasons.

## Conclusions

This first report of transovarial transmission of KFDV, and the presence of the virus in a hitherto undescribed range of vectors and habitats, will hopefully help disease managers direct their efforts for improved KFD surveillance, management and mitigation strategies and ultimately lead to communities becoming more resilient to the risk of this tick-transmitted disease.

## Supplementary Information

Additional file 1.

## Data Availability

Sequence data that support the findings of this study are available in GenBank (accession nos. PQ687485–PQ687495).
