# Supplementary material for: First evidence of transovarial transmission of Kyasanur Forest disease virus in Haemaphysalis and Rhipicephalus ticks in the wild
Source: Parasit Vectors. 2025 Jan 17;18:14. doi: 10.1186/s13071-024-06643-5 (PMC11740564; doi:10.1186/s13071-024-06643-5)
Supplement: Supplementary file 1 — Additional file 1. [file 13071_2024_6643_MOESM1_ESM.docx]

**First evidence of transovarial transmission of Kyasanur Forest disease virus in Rhipicephalus and Haemaphysalis ticks in the wild**

**Sarah J. Burthe*, Bhimanagoud Kumbar*, Stefanie M. Schäfer, Bethan V. Purse, Abi Vanak, Natrajan Balakrishnan, Richard Hassall, Subhash L Hoti, Darshan Narayanaswamy, Santoshkumar Potadar, Mujeeb Rahman and Mohammed Mudassar Chanda***

**Additional Information**

**Table S1:** Primers used for molecular identification of tick species, detection of KFDV-positive ticks and for blood meal analysis of questing larval ticks.

| **Method** | **Primer Name** | **Sequence** | **Expected product size (bp)** | **Citation for primers** |
| --- | --- | --- | --- | --- |
| Identification of tick species by molecular tools, targeting the standard COI DNA barcoding region of the the cytochrome oxidase I gene. | LCO1490 | GGTCAACAAATCATAAAGATATTGG | 709 | [[1]](https://sciwheel.com/work/citation?ids=349580&pre=&suf=&sa=0&dbf=0) |
|  | HCO2198 | TAAACTTCAGGGTGACCAAAAAATCA |  |  |
| Detection of KFDV-positive larval ticks | KFDNS5 3S | GTCAGATGAACAAAATCGCTGG | 756 | [[2]](https://sciwheel.com/work/citation?ids=4591004&pre=&suf=&sa=0&dbf=0) |
|  | KFDNS5 3R | TCATCCCCACTGACCAGCAT |  |  |
|  | KFDNS5 4S | GAAGAAGCTGTCCGAACTC | 355 |  |
|  | KFDNS5 4R | GGTCCTGTGAGTCAGATGG |  |  |
| Blood meal analysis: detection of host DNA using vertebrate-universal primers | L14724_hk3 | GGACTTATGACATGAAAAATCATCGTTG | 1140 | [[3]](https://sciwheel.com/work/citation?ids=12282080&pre=&suf=&sa=0&dbf=0)[[4]](https://sciwheel.com/work/citation?ids=13430527&pre=&suf=&sa=0&dbf=0) |
|  | H15915_hk3 | TCTCCATTTCTGGTTTACAAGAC |  |  |

**Table S2 Table of the 41 systematically sampled sites and the 8 reactive sites (end of table) giving details of the total amount of dragging and flagging transects for ticks undertaken in each habitat type.**

| Village | Houses/gardens | Arable (maize) | Non-cultivated (meadows, scrub, fallow) | Rice | Forest (dry and moist deciduous, or mixed) | Plantation coffee, tea | Plantation Acacia | Plantation Arecanut | Plantation Coconut | Plantation Eucalyptus | Plantation Rubber | Plantation Silver oak | Plantation Teak |
| --- | --- | --- | --- | --- | --- | --- | --- | --- | --- | --- | --- | --- | --- |
| Achepete | 10 |  |  | 10 | 10 |  |  | 10 |  |  |  |  |  |
| Akalapura | 10 |  |  | 11 | 12 |  |  | 9 |  |  |  |  |  |
| Bobbige | 10 |  | 10 | 10 |  |  |  | 10 |  |  |  |  |  |
| Chetalayam | 10 |  |  | 10 | 10 |  |  |  |  |  |  |  |  |
| Chulliyod | 10 |  |  |  |  | 10 |  |  |  |  |  | 10 |  |
| Dasanakodige | 10 |  |  | 5 | 3 |  |  | 14 |  |  |  |  |  |
| Demlapura | 13 |  |  | 10 | 10 |  | 10 |  |  |  |  |  |  |
| Gante Janagal | 14 |  | 2 | 8 | 10 |  |  | 10 |  |  |  |  |  |
| Guddepal | 4 |  |  | 10 | 10 |  |  | 10 |  |  |  |  |  |
| Hadigallu | 10 |  |  | 10 | 10 |  |  | 10 |  |  |  |  |  |
| Hallibailu | 10 |  |  | 10 | 10 |  |  | 10 |  |  |  |  |  |
| Kagodu | 11 |  | 10 |  | 9 |  |  | 10 |  |  |  |  |  |
| Kalpetta |  |  |  |  |  | 10 |  |  | 10 |  |  |  |  |
| Kaniyambetta |  |  | 5 | 10 | 10 | 9 |  | 5 |  |  |  |  |  |
| Kannangi | 10 |  |  | 10 | 10 |  |  | 10 |  |  |  |  |  |
| Kaudalli | 10 |  |  | 10 | 10 |  |  | 10 |  |  |  |  |  |
| Keladi | 11 |  | 10 | 8 | 6 |  |  | 5 |  |  |  |  |  |
| Kilandur | 13 |  | 7 | 1 |  |  |  | 11 |  | 3 |  |  |  |
| Mallali | 7 |  | 3 | 10 | 10 |  |  | 10 |  |  |  |  |  |
| Kodige | 14 |  |  | 10 | 10 |  |  |  |  |  |  |  |  |
| Kudumallige |  |  | 10 | 10 | 10 |  |  | 10 |  |  |  |  |  |
| Kunaji | 11 |  |  | 8 | 10 |  |  | 10 |  |  |  |  |  |
| Kunchenahalli | 10 | 10 | 2 |  | 8 |  |  | 10 |  |  |  |  |  |
| Kuppadithara | 10 |  |  | 10 |  |  |  |  |  |  | 10 |  |  |
| Malandur | 10 |  | 10 |  | 10 |  |  | 10 |  |  |  |  |  |
| Mattur | 10 |  |  | 10 |  |  |  | 10 |  |  |  |  |  |
| Meenangadi |  |  | 10 | 10 |  | 5 |  | 5 |  |  |  |  |  |
| Mullanvayal |  |  |  |  |  | 2 |  |  |  |  |  |  |  |
| Nadakalasi | 10 |  | 10 | 11 |  |  |  | 10 |  |  |  |  |  |
| Nambiarkunnu | 10 |  | 10 |  |  | 11 |  | 10 |  |  |  |  |  |
| Noolpuzha |  |  |  | 11 |  |  |  |  |  |  |  |  | 10 |
| Pallapathur |  |  |  |  | 10 |  |  |  |  |  |  |  |  |
| Poothadi |  |  |  |  |  | 10 |  |  |  |  |  |  | 20 |
| Village | Houses/gardens | Arable (maize) | Non-cultivated (meadows, scrub, fallow) | Rice | Forest (dry and moist deciduous, or mixed) | Plantation coffee, tea | Plantation Acacia | Plantation Arecanut | Plantation Coconut | Plantation Eucalyptus | Plantation Rubber | Plantation Silver oak | Plantation Teak |
| Pulpally |  |  |  | 10 |  |  |  |  |  |  | 10 |  | 10 |
| Ranjadakatte | 10 |  |  | 10 | 2 |  |  | 11 |  |  |  |  |  |
| Sasithota | 13 |  |  | 10 | 10 |  |  | 10 |  |  |  |  |  |
| Suligodu | 11 |  | 2 |  | 4 |  |  | 2 |  |  |  |  |  |
| Talale | 10 |  |  | 10 | 10 |  |  | 10 |  |  |  |  |  |
| Tanikal | 10 |  |  | 10 | 10 |  |  | 10 |  |  |  |  |  |
| Tuduru | 14 |  |  | 10 | 10 |  |  | 10 |  |  |  |  |  |
| Vakery |  |  | 10 | 10 |  |  |  |  |  |  | 10 |  |  |
| **Reactive sites** |  |  |  |  |  |  |  |  |  |  |  |  |  |
| RS Aralagodu |  |  |  |  | 5 |  |  |  |  |  |  |  |  |
| RS Kanchekai |  |  | 2 |  | 8 |  |  |  |  |  |  |  |  |
| RS Marbedi | 10 |  |  | 11 | 11 |  |  | 10 |  |  |  |  |  |
| RS Mathikere | 2 |  |  |  |  |  |  | 6 |  |  |  |  |  |
| RS MD Bidure |  |  |  |  | 2 |  |  |  |  |  |  |  |  |
| RS Nallimakki | 1 |  |  |  |  |  |  |  |  |  |  |  |  |
| RS Sampa |  |  |  |  |  |  |  | 4 |  |  |  |  |  |
| RS Vatemakki | 5 |  |  |  |  |  |  |  |  |  |  |  |  |

**Table S3: Numbers of larvae sampled for each of the five species with KFDV positive questing larvae, indicating the total number of transects with both positive and negative larvae.**

| Species | Total number of larvae | Total transects with negative larvae | Total transects with positive larvae |
| --- | --- | --- | --- |
| *Haemaphysalis bispinosa* | 474 | 122 | 2 |
| *Haemaphysalis spinigera* | 801 | 154 | 3 |
| *Rhipicephalus microplus* | 388 | 87 | 3 |
| *Rhipicephalus annulatus* | 39 | 10 | 2 |
| *Haemaphysalis* sp. 01 (no close GenBank match) | 171 | 40 | 1 |

**Molecular identification of the larval tick specimens.**

The standard DNA barcoding region of the mitochondrial COI gene was amplified and birectionally sequenced using universal primer pair LCO1490 (5’-GGTCAACAAATCATAAAGATATTGG-3’) and HCO2198 (5’-TAAACTTCAGGGTGACCAAAAAATCA-3’) [[5]](https://sciwheel.com/work/citation?ids=246600&pre=&suf=&sa=0&dbf=0). Raw forward and reverse reads were edited in SeqMan® (version 17.6. DNASTAR. Madison, WI), creating 658bp COI DNA barcodes. These were compared with public data deposited in the NCBI GenBank using the BLAST tool. GenBank deposits with above 97% sequence identity were downloaded, and, together with our COI barcodes, used to create multiple sequence alignments in BioEdit (version 7.2.6). The final alignment, containing a subset of GenBank downloads, was analysed in MEGA (version 11.0.13) and using the UMPGA (unweighted pair group method with arithmetic mean) method to depict the phylogenetic clustering of the tick COI barcodes.

Both the BLAST searches as well as phylogenetic analyses show that the GenBank data depository contains numerous COI barcodes of tick specimen that have been mis-identified morphologically. This is the case for two GenBank submitted COI sequences that are the only close matches to our KFDV-positive unfed larva No 13 (Haemaphysalis_sp_01_L13_Demplapura_HOUSE_GARDEN), which could not be ID ed morphologically to species level: sequences with accession number OR268624 (described as “Ixodidae sp. isolate *Haemaphysalis leporispalustris*”) and OR268625 (“Ixodidae sp. isolate *Haemaphysalis danieli*”) are both near identical to each other as well as our larva No 13 (>99.5% identity, 85%-91% query cover) but show less than 90% sequence identity to any other GenBank submissions, with the clostest matches being numerous *H. concinna* deposits (n=50, 88%-89% identity) and showing less than 85% sequence identity to any of the *H. danieli* or *H. leporispalustris* GenBank COI deposits.

**Figure S1: Phylogenetic tree based on tick COI DNA barcodes.** The names of the COI barcodes generated from the KFDV-positive unfed larvae collected are given as: Species_Sample No_Sampling site_Habitat type. The labels for the GenBank downloads are bearing a “gb” at the beginning and the GenBank accession number at the end. The evolutionary history was inferred using the UPGMA method [[6]](https://sciwheel.com/work/citation?ids=16944058&pre=&suf=&sa=0&dbf=0). The optimal tree is shown. The percentage of replicate trees in which the associated taxa clustered together in the bootstrap test (1000 replicates) are shown next to the branches [[7]](https://sciwheel.com/work/citation?ids=4076711&pre=&suf=&sa=0&dbf=0). The tree is drawn to scale, with branch lengths in the same units as those of the evolutionary distances used to infer the phylogenetic tree. The evolutionary distances were computed using the Maximum Composite Likelihood method [[8]](https://sciwheel.com/work/citation?ids=1106418&pre=&suf=&sa=0&dbf=0) and are in the units of the number of base substitutions per site. This analysis involved 28 nucleotide sequences. Codon positions included were 1st+2nd+3rd+Noncoding. All ambiguous positions were removed for each sequence pair (pairwise deletion option). There were a total of 658 positions in the final dataset.


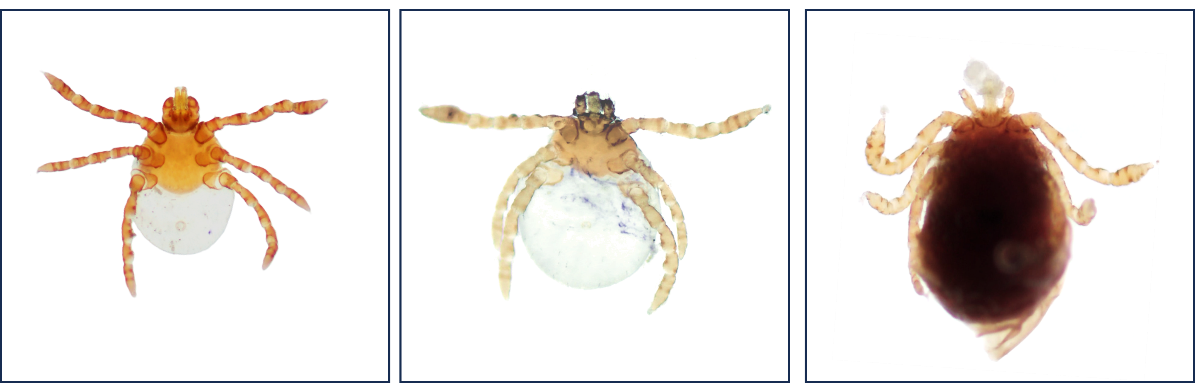


**Figure S2:** Photographs of larvae comparing the idiosoma of an unfed questing larva collected by drag or flag sampling (left-hand panel, no evidence of a blood meal) with two partially engorged larvae (middle and right-hand panel) taken from a vertebrate host and which have had a blood meal.


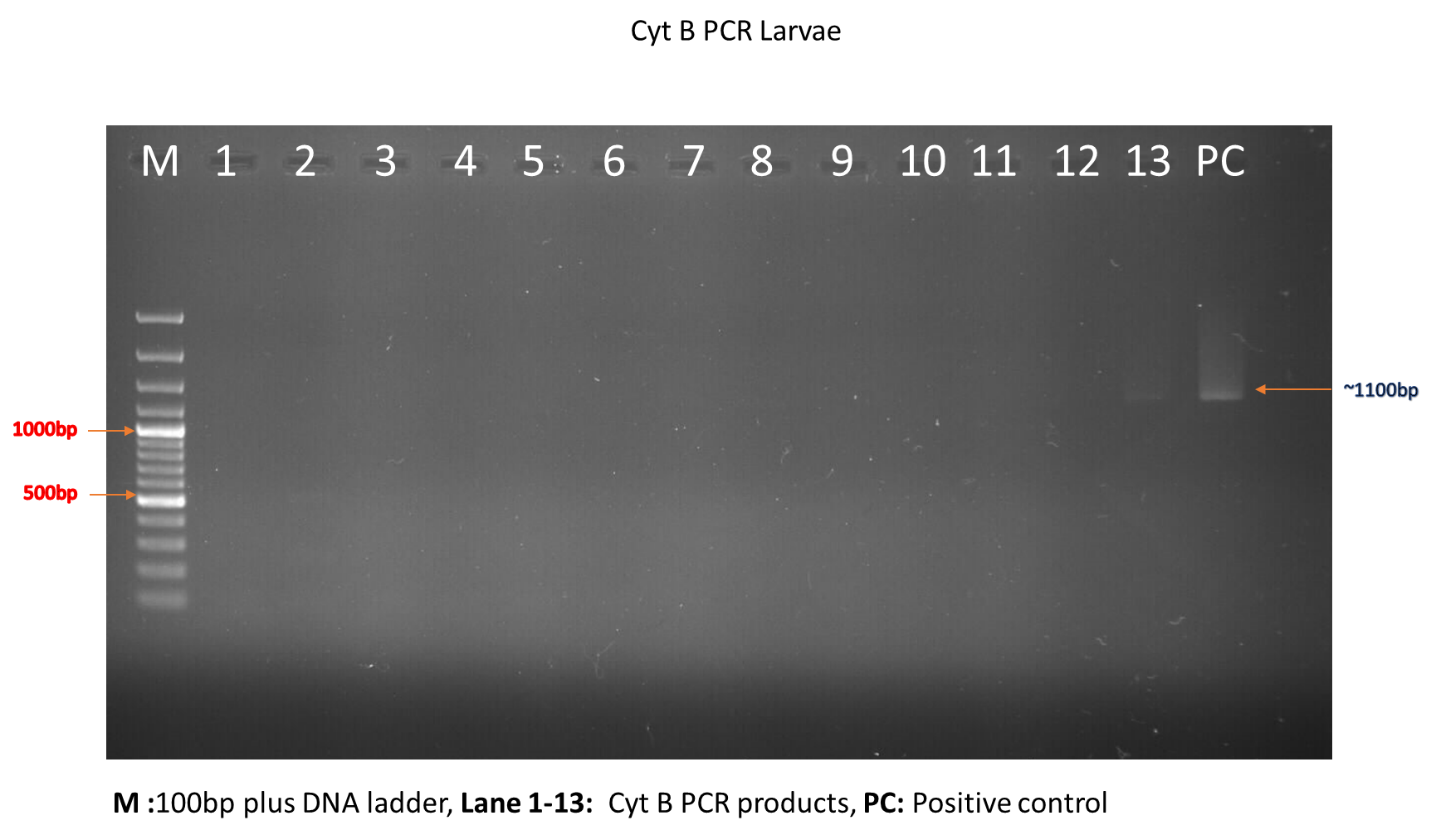


**Figure S3:** Gel results of the blood meal analysis indicating that none of the larval ticks collected from the environment (samples number 1 to 12) showed any evidence of vertebrate DNA that would be indicative of these ticks having received a blood meal. The larval tick in lane 13 was included as a further positive control and was from a larval tick that was removed from a shrew (*Suncus montanus*) host in a partially fed state.


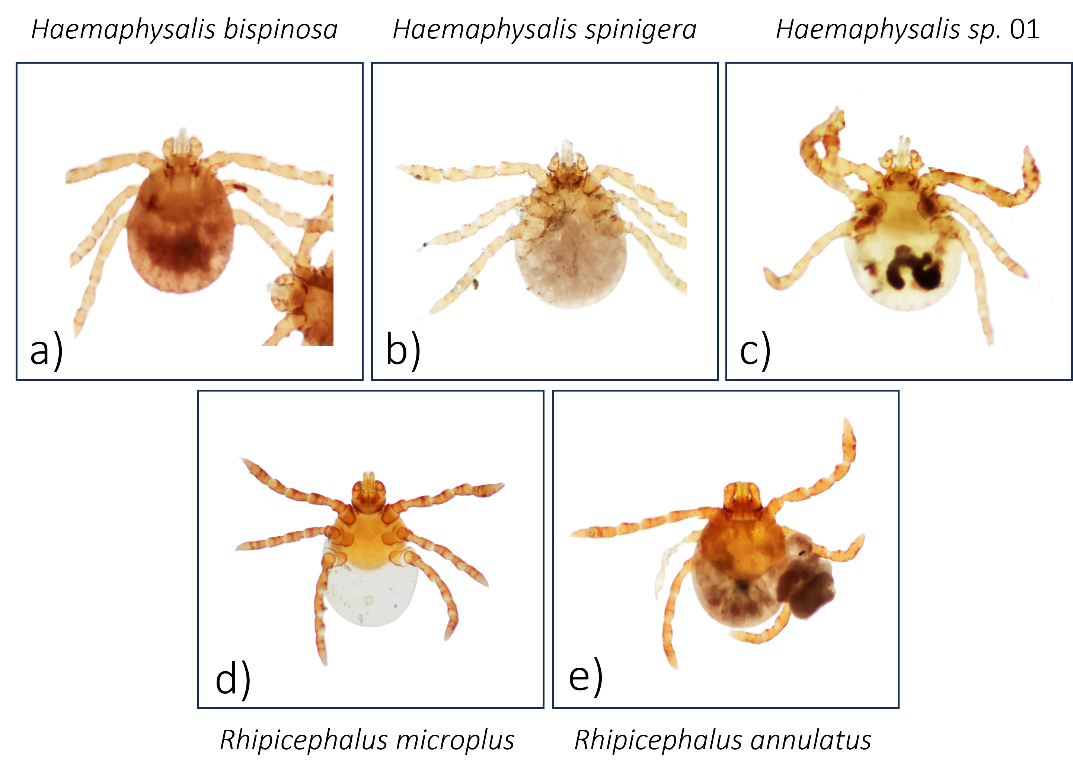


**Figure S4:** Larval tick specimens from the five different species identified that were positive for KFDV, photographed at 5x magnification using a stereomicroscope (Zeiss^TM^): a) *Haemaphysalis bispinosa*; b) *H. spinigera;* c) *Haemaphysalis* spp. (no close match in Genbank; referred to as Haemaphysalis_sp_01_L13 in Fig. S1); d) *Rhipicephalus microplus*; and e) *R. annulatus.*

**References**

[1. Folmer O, Black M, Hoeh W, Lutz R, Vrijenhoek R. DNA primers for amplification of mitochondrial cytochrome c oxidase subunit I from diverse metazoan invertebrates. Mol Marine Biol Biotechnol. 1994;3:294–9.](https://sciwheel.com/work/bibliography/349580)

[2. Mourya DT, Yadav PD, Mehla R, Barde PV, Yergolkar PN, Kumar SRP, et al. Diagnosis of Kyasanur forest disease by nested RT-PCR, real-time RT-PCR and IgM capture ELISA. J Virol Methods. 2012;186:49–54.](https://sciwheel.com/work/bibliography/4591004)

[3. He K, Li Y-J, Brandley MC, Lin L-K, Wang Y-X, Zhang Y-P, et al. A multi-locus phylogeny of Nectogalini shrews and influences of the paleoclimate on speciation and evolution. Mol Phylogenet Evol. 2010;56:734–46.](https://sciwheel.com/work/bibliography/12282080)

[4. Zhang B, He K, Wan T, Chen P, Sun G, Liu S, et al. Multi-locus phylogeny using topotype specimens sheds light on the systematics of Niviventer (Rodentia, Muridae) in China. BMC Evol Biol. 2016;16:261.](https://sciwheel.com/work/bibliography/13430527)

[5. Hebert PD, Cywinska A, Ball SL, deWaard JR. Biological identifications through DNA barcodes. Proc Biol Sci. 2003;270:313–21.](https://sciwheel.com/work/bibliography/246600)

[6. James MT, Sokal RR, Sneath PHA. Numerical Taxonomy. Evolution. 1964;18:513.](https://sciwheel.com/work/bibliography/16944058)

[7. Felsenstein J. Confidence limits on phylogenies: an approach using the bootstrap. Evolution. 1985;39:783–91.](https://sciwheel.com/work/bibliography/4076711)

[8. Tamura K, Nei M, Kumar S. Prospects for inferring very large phylogenies by using the neighbor-joining method. Proc Natl Acad Sci USA. 2004;101:11030–5.](https://sciwheel.com/work/bibliography/1106418)
